# Extreme strength observed in limpet teeth

**DOI:** 10.1098/rsif.2014.1326

**Published:** 2015-04-06

**Authors:** Asa H. Barber, Dun Lu, Nicola M. Pugno

**Affiliations:** 1 School of Engineering, University of Portsmouth, Portsmouth PO1 3DJ, UK; 2 Laboratory of Bio-Inspired and Graphene Nanomechanics, Department of Civil, Environmental and Mechanical Engineering, Università di Trento, via Mesiano, 77, 38123 Trento, Italy; 3 Center for Materials and Microsystems, Fondazione Bruno Kessler, Via Sommarive 18, 38123 Povo (Trento), Italy; 4 School of Engineering and Materials Science, Queen Mary University of London, Mile End Road, London E1 4NS, UK

**Keywords:** mechanics, nanoscale, mineralized tissue

## Abstract

The teeth of limpets exploit distinctive composite nanostructures consisting of high volume fractions of reinforcing goethite nanofibres within a softer protein phase to provide mechanical integrity when rasping over rock surfaces during feeding. The tensile strength of discrete volumes of limpet tooth material measured using *in situ* atomic force microscopy was found to range from 3.0 to 6.5 GPa and was independent of sample size. These observations highlight an absolute material tensile strength that is the highest recorded for a biological material, outperforming the high strength of spider silk currently considered to be the strongest natural material, and approaching values comparable to those of the strongest man-made fibres. This considerable tensile strength of limpet teeth is attributed to a high mineral volume fraction of reinforcing goethite nanofibres with diameters below a defect-controlled critical size, suggesting that natural design in limpet teeth is optimized towards theoretical strength limits.

## 1. Introduction

**Figure 1.**
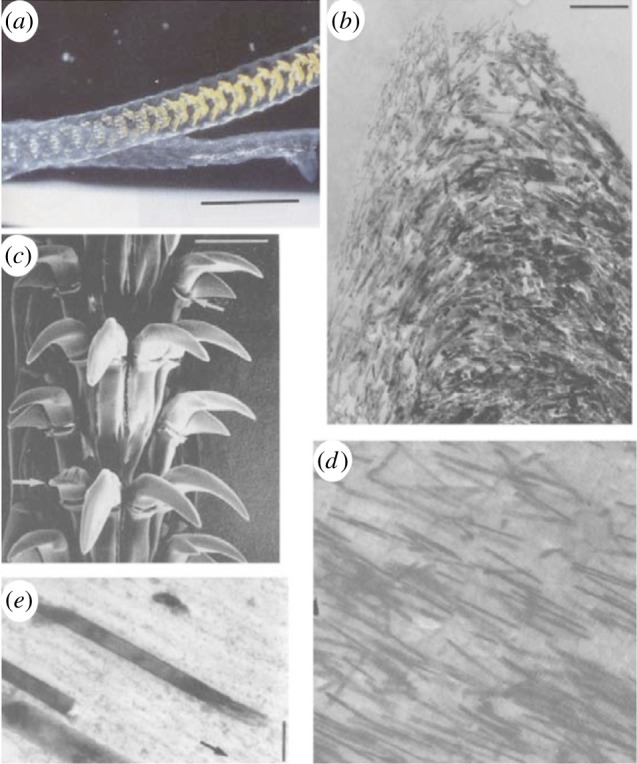
Structure of the common limpet tooth (*Patella vulgata*). (*a*) Optical image of the tongue-like radula containing bands of teeth along a length of many centimetres. (*b*) Scanning electron micrograph of the teeth groupings with each tooth length approximately 100 μm. High-magnification electron microscopy images of the tooth cusp show (*c*) the changing orientation of the nanofibrous goethite in the chitin matrix and (*d*) the high anisotropy of the composite at the anterior and posterior edges owing to alignment of the goethite, note the mineral fibre length of approx. 3 μm, with (*e*) close-up of the tooth indicating the distinct phases of the goethite ‘reinforcing fibre’ and the chitin ‘matrix’ highlighting the structural resemblance to a fibre-reinforced composite material with an average fibre diameter of approx. 20 nm. Adapted from reference [12]. (Online version in colour.)

Composite structures are widespread in nature and are ubiquitous in mineralized tissue where protein-based polymer frameworks are reinforced with a stronger and stiffer mineral phase [1,2]. These composite structures often have a distinct mechanical function and have led to a number of engineering principles being applied to explain resultant structure–function behaviour in biological organisms [1–4]. More recent concepts have examined the potential of biology in controlling the size of constituents in natural composite structures particularly for enhanced mechanical properties at small length scales. Specifically, the reinforcing mineral phase in many organisms approaches nanometre length scales, at least in one-dimension, which has been proposed as promoting flaw insensitivity to increase the tensile strength of mineralized tissue [5]. The enhancement of material tensile strength owing to size effects has additionally been shown historically, including Griffith's observations of increased glass fibre failure stress as their diameters decreased [6], to more recent quantized fracture mechanics (QFM) extensions [7] from statistical descriptions of material strength by Weibull [8]. However, the insensitivity of materials to flaws has been shown to operate at length scales of many tens of nanometres [5], so that material failure is governed by the theoretical strength of the material and not by stress concentrations around flaws as first considered by Griffith [6]. Discrete examples of exceptional strength in natural materials are perhaps most prevalent in the silk of spiders [9,10], with tensile strength values of up to 4.5 GPa recorded in the literature [11]. Limpet teeth shown in figure 1 are an example of a material produced biologically that is optimized for strength, especially as these teeth need to be extremely strong and hard to avoid catastrophic failure when rasping over rock surfaces during feeding.

Recent work has shown that the teeth of limpets approximate to an almost ideal model natural composite material where high aspect ratio mineral nanofibres of goethite reinforce a protein matrix [13]. Limpet teeth are also notable by displaying a lack of structural hierarchy present in many other mineralized tissue structures. As structural hierarchy has been shown to dictate resultant natural composite material strength [14], limpet teeth are particularly relevant for the examination of size-dependent tensile strength in natural materials without the influence of additional structural features across a range of length scales. The composition of limpet teeth consists primarily of mineral nanofibres typically many micrometres in length but only a few tens of nanometres in diameter, thus below the critical size defined as promoting flaw insensitivity [5], and occupy a significant volume fraction of approximately 80% in mature teeth [13]. The strength of the limpet tooth must therefore be critically dependent on the strength of the mineral nanofibres within the composite structure. Indeed, the density of flaws in the reinforcement phase has been previously shown to define the tensile strength of engineered composite materials containing fibres with diameters that are over two orders of magnitude larger than the nanofibres found in limpet teeth [15,16]. Limpet teeth therefore present a natural structure with the potential to optimize composite strength towards a theoretical maximum by the incorporation of nanofibre constituents below a critical size that defines tolerance to flaws [5].

Considerable challenges exist in measuring the tensile strength of limpet teeth, and indeed, any mineralized tissue, owing to difficulties in separating the influence of this material behaviour from structural organization. Such challenges have led to strategies where discrete volumes of material are first isolated from the parent sample and subsequently mechanically tested. The selection of a discrete material volume is driven by the need to simplify the structure such as the removal of structural hierarchy or examination of a specific structural orientation when evaluating mechanical properties. Focused ion beam (FIB) microscopy has been demonstrated as a powerful technique in isolating discrete material volumes for mechanical testing in mineralized tissue, including teeth and bone [13,17,18], as well as examining the fracture toughness at interfaces in metals and alloys [19,20]. Previous work from our group has highlighted the effectiveness of isolating rectangular beams with widths approaching 1 μm using FIB methods, so that the resultant sample approximates towards a uniaxially aligned short fibre composite [13]. Corresponding mechanical testing of mineralized tissue at small length scales is measured by atomic force microscopy (AFM) and has been proved to be effective for the measurement of the elastic properties of limpet teeth [13] and bone [18] as well as the failure strength of human teeth [17]. However, tensile testing along the principal structural axis of discrete material volumes such as defined by the orientation of the reinforcing mineral nanofibres in limpet teeth is yet to be achieved. AFM has been applied to uniaxial testing of individual nanofibrous materials, including mineralized collagen fibrils [21], polymer nanofibres [22] and nanotubes [23,24], and relies on integration of AFM with scanning electron microscopy (SEM) in order to manipulate and observe relatively small material volumes for tensile testing. Therefore, AFM techniques show suitability in assessing the strength of limpet teeth in terms of their material behaviour and are applied in this work as a novel experimental technique to determine the tensile strength of discrete limpet teeth volumes.

## 2. Material and methods

### 2.1. Sample preparation

Samples of the limpet *Patella vulgata* were harvested in Southampton, UK and fixed in seawater prior to transportation to the laboratory. The limpets were immediately rinsed in running tap water in the laboratory and sacrificed during storage in a refrigerator held at −15°C. Teeth were isolated by first removing the limpet from storage and holding at room temperature for 3 h, thus allowing thawing of the organism. The tongue-like radula appendage containing limpet teeth was dissected from the visceral mass of the limpet under an optical microscope and stored in 80% ethanol. The radula end containing the first 5–10 rows of teeth showed evidence of wear from rasping over rock surfaces during feeding and was removed using dissection. The remaining radula length was cut into sections with approximate lengths ranging from 3 to 7 mm and mounted onto a standard electron microscope aluminium stub using carbon tape. The radula was then placed on the stub in a drop of water and manipulated, so that the radula length was extended and did not curve or roll up using fine needles while observing under an optical microscope. The radula was allowed to dry on the surface of the carbon tape fixed onto the aluminium stub. Silver paint was further applied to the base of the tooth to suppress charge accumulation during the electron and ion beam microscopy. Small dimension limpet tooth samples were prepared using FIB techniques previously applied to mineralized tissue of bone [18]. Fabrication of small limpet tooth samples was achieved using an experimental system containing AFM (attocube, Germany) integrated within a dual-beam instrument (Quanta 3D, FEI, USA/EU) to provide patterning, manipulation and *in situ* observation. A FIB-flattened AFM probe (Veeco, USA, spring constant of 200 N m^−1^) was first translated into glue (Poxipol, Arg.) contained within the SEM chamber to allow glue pick-up at the end of the probe. The AFM tip containing the glue was subsequently moved towards the cusp apex of the limpet tooth while monitoring using the secondary electron imaging of the SEM as shown in figure 3*a*. After subsequent curing of the glue after 1 h, FIB was used to section the limpet tooth as indicated in figure 3*b* using gallium ions accelerated at 30 kV and an ion current of 1 nA. Sample ‘dog-bone’ geometries were achieved using further FIB sectioning to remove limpet tooth material.

### 2.2. Mechanical fragmentation testing

**Figure 2.**
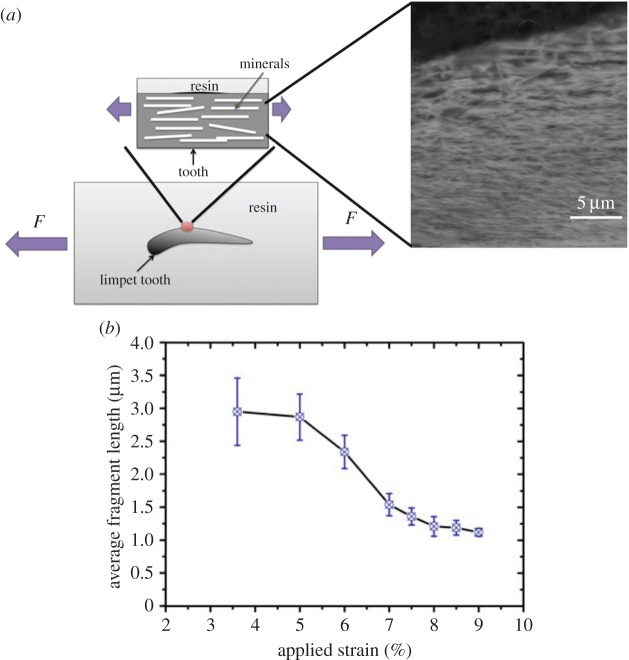
Failure of the limpet teeth structure was achieved by (*a*) embedded teeth in an epoxy resin and tensile testing to failure. Backscattered SEM images clearly indicated the nanofibrous structure. (*b*) Plot of the average length of the nanofibres during straining of the teeth embedded in epoxy resin, indicating failure of the nanofibre reinforcement that cause a fragmentation of the nanofibres to smaller lengths. (Online version in colour.)

Limpet teeth were dissected from the radula and mixed with epoxy resin (Poxipol, Arg.) and allowed to stand for 1 h to allow the glue to fully cure. Examination of the epoxy resin in SEM showed a number of individual teeth partially exposed at the top surface of the resin. The cured specimens were cut to a thickness of 2 mm, length of 10 mm and width of 4 mm using a diamond saw. The epoxy resin top surface containing the limpet teeth was mechanically polished opposite to the surface containing limpet teeth. FIB was used to further polish the surface of the limpet tooth, so that goethite nanofibre minerals were easily detectable at this surface as shown in figure 2*b* using SEM in backscattered imaging mode to give high contrast between the nanofibres and chitin matrix. Samples were submerged in seawater for 24 h and immediately transferred to a tensile testing machine (Instron, USA) with a 1 kN load cell. Mechanical testing of the epoxy resin containing limpet teeth was carried out to failure at a displacement rate of 0.1 mm per minute to produce fragmentation of the mineral nanofibres. Samples were removed from the tensile tester at various strain values and the anterior edge of limpet tooth imaged using low vacuum SEM. Analysis of the average nanofibre length was carried out using ImageJ (NIH, USA), with the average nanofibre length at the tooth's anterior edge shown to decrease with applied strain as shown in figure 2*b*.

### 2.3. Nanomechanical testing

**Figure 3.**
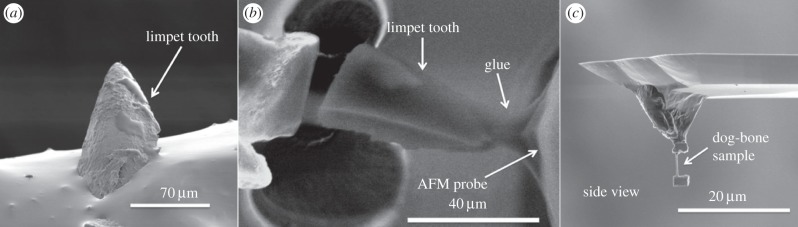
Scanning electron micrographs showing (*a*) the limpet tooth prior to FIB milling, (*b*) FIB sectioning and attachment of the limpet tooth cusp to an AFM probe and (*c*) further FIB milling to thin the sample towards a ‘dog-bone’ geometry.

Mechanical testing of the FIB-fabricated limpet tooth samples was achieved using a custom-built AFM–SEM set-up [18,21,22]. We note that this set-up provides a hydrated environment to a range of materials within a 2 h window of opportunity. Tensile testing was achieved by translating the free end of the FIB-prepared ‘dog-bone’ limpet tooth sample attached to the AFM into a second droplet of wet glue introduced to the AFM sample stage within the SEM chamber. The glue was allowed to fully cure after 1 h in the SEM chamber to produce a tensile test configuration as shown in figure 3. Tensile testing was achieved by translating the AFM tip away from the second glue droplet surface at a rate of 1 μm s^−1^ using the piezo positioners of the AFM, which caused deformation of the sample until failure. *In situ* SEM imaging allowed observation of glue displacement, and none was observed in successful mechanical tests. The stress and strain of the sample was calculated using a fibre optic situated behind the AFM cantilever in order to determine the cantilever deflection during sample deformation. The sample displacement *d* was calculated using *d* = *Z* − *δ*, where *Z* is the piezo displacement and *δ* is the AFM cantilever deflection measured from the fibre optic. Strain in the sample *ɛ* was therefore calculated using *ɛ* = *d*/*L*, where *L* was the initial sample length measured from SEM imaging. The stress in the sample *σ* was calculated from *σ* = *F*/*A*, where *A* was the cross-sectional area of the sample and *F* was the force applied to the sample. This area *A* was measured from SEM imaging, and the error is lower than 5%. Similarly, the force *F* was determined by recording the AFM cantilever deflection during sample deformation and knowing the spring constant of the AFM cantilever, calibrated using the thermal noise method [25]. The error in force measurements using this calibration method has been shown to be lower than 5% [26]. Thus, the error in the strength cannot be larger than 11%. Sample drift during curing of the glue prior to the tensile test was found to cause misalignment or premature sample failure, resulting in approximately one in every 10 samples prepared being successfully tensile tested to failure in this work.

## 3. Results

Failure of limpet tooth was first evaluated using a macroscopic fragmentation test to establish the failure behaviour of the material and qualitatively justify failure of the reinforcing mineral phase in the tooth. Limpet teeth embedded within a solid epoxy resin were prepared, as shown in figure 2, for tensile testing. A polished sample surface clearly exhibited the nanofibrous goethite as observed under backscattered electron imaging. Tensile testing of hydrated samples caused a progressive failure of the nanofibres, defined as a fragmentation of the nanofibres, resulting in a reduction in the average nanofibre length with applied tensile strain as shown in figure 2*b*. Fragmentation of the reinforcing phase is established in composites evaluations, with the progressive reduction in reinforcing fibre lengths until a plateaux region indicative of a stress ‘saturation’ [27]. This fragmentation of the reinforcing mineral phase therefore confirms that stress transfer within the tooth material is sufficient to fail the goethite nanofibres, as opposed to potential interfacial failure and pull-out of the nanofibrous phase commonly encountered in tough biological materials [21]. Thus, the failure of mineral phase defines the limpet tooth as a potentially strong material. Evaluating the failure of limpet teeth then progressed to mechanical testing of discrete material volumes.

**Figure 4.**
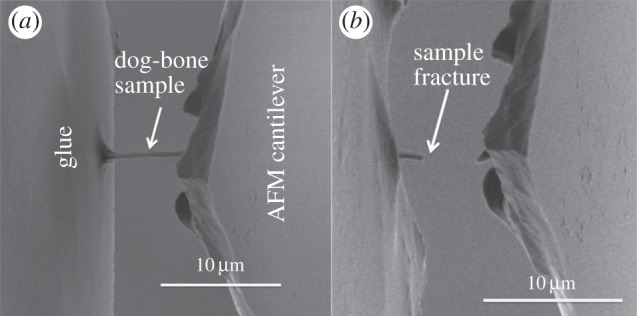
Scanning electron micrograph of (*a*) the limpet tooth sample attached to the AFM cantilever set-up and partially embedded within gripping glue and (*b*) failure at the sample free length mid-point after tensile testing.

**Figure 5.**
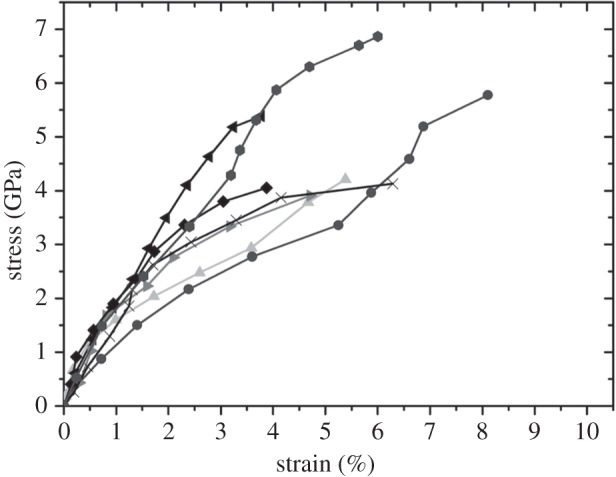
Plot of the stress–strain behaviour of individual limpet tooth samples, with a variety of lengths, tensile tested to failure using AFM.

**Figure 6.**
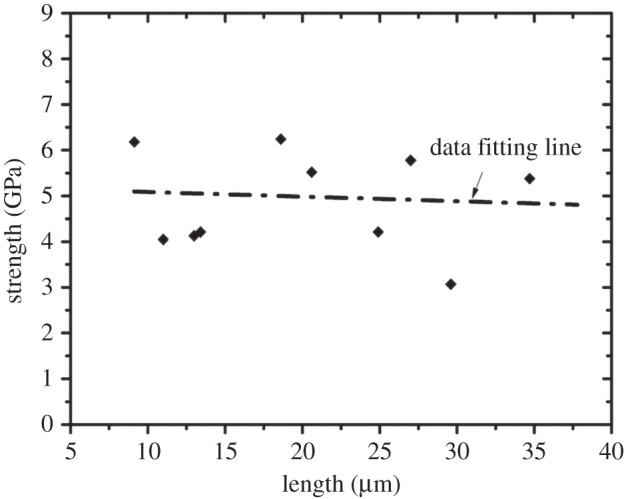
Plot of the tensile strength of limpet tooth material with varying sample length. The data fitting line shows the general trend of minimal strength variation with sample length.

Isolation of discrete volumes of limpet tooth from the cusp region of the tooth, using FIB to produce the sample, as well as manipulation to attach the sample to an AFM probe, is shown in figure 3. FIB was particularly effective in producing a sample approaching conventional larger scale ‘dog-bone’ type geometries where the sample volume of interest is relatively long and narrow, whereas a larger amount of material at either end of the sample enhances gripping during mechanical deformation. The dog-bone sample was tensile tested to failure using AFM while observing with SEM as shown in figure 4. The stress–strain behaviour of the limpet tooth samples was recorded during tensile testing until failure. Figure 5 shows a plot of the variation in limpet tooth stress as the sample was strained to failure. All samples showed a pronounced linear elastic behaviour until failure, with evidence of nonlinearity when the sample strain exceeded approx. 2%. Variability in the stress–strain curves is observed, because the distribution of the reinforcing phase within the limpet tooth is not particularly highly ordered as shown in figure 1*d*. For example, a higher elastic modulus can be expected owing to the discrete volume tested containing a relatively large amount of mineral phase compared with other samples. The deformation of the tooth can be explained by consideration of the corresponding structure tested. Limpet teeth contain a high volume fraction of mineral phase, with the stress–strain response expected to be dominated by the mechanical properties of the reinforcing mineral. The softer protein matrix will potentially contribute to the nonlinear stress–strain behaviour, especially as the goethite phase present in the tooth has been shown to be linear elastic [28]. However, the linear elastic modulus of the tensile tested limpet tooth samples taken from the plot in figure 5 is 120 ± 30 GPa, which is beyond an expected polymeric value and approaches elastic moduli values of around 180 GPa measured for the pure mineral phase [28]. Thus, the deformation behaviour of the limpet teeth is justified as being dominated by the mineral phase. The maximum tensile stress at failure of the limpet tooth samples in figure 5 shows variability within the dataset. Variability in the strength of the limpet tooth material is defined by either the length of the sample tested, as described by evaluations of stress concentrations around flaws [6–8,11,13], or governed by flaw insensitivity of the mineral nanofibres [5]. A plot of the tensile strength of the limpet tooth samples is shown in figure 6 as the length of specimen tested is varied. The strength of the limpet tooth samples, defined by the mineral phase, lies within a range from 3.0 to 6.5 GPa and again confirms that the tensile strength of a limpet tooth is potentially the highest ever recorded in nature, exceeding the strength of spider silk fibres [11]. A simple mean line fitted through the experimental data in figure 6 indicates a lack of size-dependent strength. Specifically, flaw density considerations dictate that the strength of a material will decrease as the gauge length of the material increases [6–8], whereas flaw tolerance is suggested as being active below a critical size [5]. The results in figure 6 therefore show that limpet teeth material shows tolerance to flaws as proposed by previous work [5].

## 4. Discussion

The tensile strength of limpet tooth samples shown in figure 6, and the corresponding elastic modulus taken from figure 4 are 4.90 ± 1.90 and 120 ± 30 GPa, respectively. The mechanical properties of limpet tooth samples are governed by deformation and breaking of chemical bonds within the goethite mineral nanofibres, and therefore can be defined from structural information on goethite. Crystallographic studies of goethite have been carried out both theoretically [28] and experimentally [29], with deformation and failure owing to the external loading axis in tensile tests predominantly aligned with the long *c*-axis of the mineral crystal. This considerable strength of limpet teeth defined from the crystal structure of goethite is potentially outstanding. As a biological comparison, the tensile strength and elastic modulus of spider silk, the material currently consider to be the strongest in nature, can reach values of up to 4.5 and 10 GPa, respectively [11], which is considerably lower than the mechanical performance of the limpet tooth. We note that limpet teeth are hybrid materials consisting of an organic and inorganic phase, whereas spider silk is exclusively organic. More recent work has used atomistic simulations to evaluate tensile mechanical properties of cellulose nanocrystals [30], with a recorded strength of just over 4 GPa still below the values of limpet tooth strength in this work. Indeed, the mechanical strength of the limpet tooth is comparable to that of the strongest man-made fibres, e.g. high-performance Toray T1000G carbon fibres have a tensile strength of 6.5 GPa. Additional comparisons could be made by consideration of high volume fraction reinforcement composites using layer-by-layer [31,32] and freeze-casting techniques [33,34]. In these cases, the composite material strengths are hundreds of MPa, highlighting the order of magnitude enhancement in tensile strength and general extreme mechanical performance of the limpet tooth samples. The lack of a significant change in the tensile strength of limpet tooth samples in figure 6, despite a fourfold increase in the length of the sample, suggest the mineral phase within the limpet tooth is below a flaw tolerant critical size of approximately 30 nm, as reported in previous literature [5]. The mineral phase may therefore operate towards a theoretical maximum limit, as suggested by the classical estimation of the theoretical strength of the composite, of the order of elastic modulus *E*/(10–30) approximately 4–12 GPa, that is compatible with our observations and is indeed suggested in materials ranging from polymeric fibres [35] to the nacreous layer in shells [36].

## 5. Conclusion

We show that the tensile strength of limpet teeth can reach values higher than spider silk, considered currently to be the strongest biological material, and only comparable to the strongest commercial carbon fibres. We have also proved that the strengths, in our investigated range, are relatively size-independent using small-scale *in situ* tensile testing. Limpet teeth structures therefore highlight the efficiency of biological control in assembling a composite structure of nanofibrous goethite for optimal strength behaviour. The goethite nanofibres are expected to dictate the flaw tolerance of the resultant composite owing to their diameters being below a critical threshold value of the order of tens of nanometres. This work demonstrates a high-strength composite found in nature and highlights a design strategy towards strong, engineered composites reinforced with a high volume fraction of nanofibrous material. As the limpet tooth is effective at resisting failure owing to abrasion, as demonstrating during rasping of the tooth over rock surfaces, corresponding structural design features are expected to be significant for novel biomaterials with extreme strength and hardness, such as next-generation dental restorations.
